# Screening of Dementia

**DOI:** 10.1155/2014/529419

**Published:** 2014-12-08

**Authors:** Rajka M. Liscic, Görsev G. Yener, Huali Wang, Jong-Ling Fuh, Jianjun Jia, Yuan-Han Yang

**Affiliations:** ^1^Institute for Medical Research and Occupation Health, Zagreb, Croatia; ^2^Department of Neurology, County Hospitals of Altötting-Burghausen, Altötting, Germany; ^3^Department of Neurology, Dokuz Eylül University, Izmir, Turkey; ^4^Dementia Care and Research Center, Peking University Institute of Mental Health, Beijing, China; ^5^Department of Neurology, Neurological Institute, Taipei Veterans General Hospital, Taipei, Taiwan; ^6^Department of Geriatric Neurology, Chinese People's Liberation Army General Hospital, Beijing, China; ^7^Department of Neurology, Kaohsiung Medical University Hospital and Kaohsiung Municipal Ta-Tung Hospital, Kaohsiung Medical University, Kaohsiung, Taiwan

Asia is the most populous region in the world and its rapidly growing societies are the sources of global development. However, aging of its population with increasing occurrence of diseases, of which dementia is the most prominent, is a major challenge to healthcare system. For example, there are 177 million people aged 65 and older living with dementia in China, expressing 20% of dementia patients worldwide. It has been estimated that the Chinese proportion of the elderly will reach 30.4% in 2050, which will include 100 million elderly people over 80 years of age [1]. Dementia prevalence in Asia, however, has previously been found to be lower than in Western populations [2]. Cultural differences could contribute to this. Dementia research in Chinese population has been primarily focused on Alzheimer's disease and vascular dementia [2]. Most of our knowledge about dementia, however, comes from studies in Caucasian population. Early and accurate diagnosis of dementia is crucial in order to start with the treatment as early as possible. Intervention and treatment of dementia (AD-dementia) can be cost-effective, but the majority of patients are not diagnosed in a timely manner. Technology is now available that can enable earlier detection of cognitive loss associated with incipient dementia, offering the potential for earlier intervention and health care systems and resulting in a less financial burden to an individual and a society. In this special issue on screening of dementia, we focused on screening tests for the detection of very mild dementia. We have invited a few papers that address those topics accordingly.

One paper of this special issue gives a view on dementia screening in an outpatient department of a regional hospital in Taiwan by means of AD8 (ascertainment of dementia 8), a brief informal interview to screen dementia [3]. Screening people at the risk of dementia is a first and a major issue in screening of dementia. Another paper explores depression as a crucial public health issue in Taiwan. By means of brief tool, Epidemiological Studies Depression Scale (CES-D), to screen depression, a ratio of 16.4% of suspected depression patients compared to 13.3% aged patients out of all recruited patients was shown. This result may provide important information for a public health issue. In another paper, by means of AD8 consistent problems with thinking and/or memory were found in 56.8% participants, difficulty in remembering appointments was found in 47% participants, forgetting correct month or year was found in 40.9% of recruited participants in Taiwan, accordingly. Another paper presents the utility of informant AD8 for case finding of cognitive impairment in primary healthcare setting in Singapore. On a sample of 205 patients and their informants, AD8 was shown to be useful for case finding of cognitive impairment in the primary healthcare in one-third of adult patients. Another paper shows efficacy of the Takeda Three Colors Combination (TTCC) test, a screening tool for detection of very mild AD-dementia in Japan. Despite a lower sensitivity, a TTCC test was accomplished within 2 minutes in all subjects, thus being a great potential for the use as an AD screening tool by general practitioners in communities worldwide. Another paper assesses the influence of education on the performance of Chinese version of the Montreal Cognitive Assessment (C-MoCA) compared to Mini-Mental State Examination (MMSE) in detecting amnestic mild cognitive impairment (aMCI) among rural population in Beijing community. C-MoCA showed modest accuracy and was no better than MMSE in detecting aMCI, most likely due to overwhelming effect of education relative to aMCI diagnosis on variations in C-MoCA performance. Finally, all presented cognitive tests show great potential for the use as screening tools for early and very mild dementia worldwide, being easy to apply and at a low cost in communities worldwide.

*Rajka M. Liscic* *Rajka M. Liscic* *Görsev G. Yener* *Görsev G. Yener* *Huali Wang* *Huali Wang* *Jong-Ling Fuh* *Jong-Ling Fuh* *Jianjun Jia* *Jianjun Jia* *Yuan-Han Yang* *Yuan-Han Yang*
